# Self-Reported Dietary Restrictions and Dietary Patterns in Polish Girls: A Short Research Report (GEBaHealth Study)

**DOI:** 10.3390/nu8120796

**Published:** 2016-12-19

**Authors:** Grzegorz Galinski, Marta Lonnie, Joanna Kowalkowska, Lidia Wadolowska, Jolanta Czarnocinska, Marzena Jezewska-Zychowicz, Ewa Babicz-Zielinska

**Affiliations:** 1 Department of Human Nutrition and Hygiene, Poznan University of Life Sciences, Wojska Polskiego 28, 60-637 Poznan, Poland; gregoryg@up.poznan.pl (G.G.); jotcezet@up.poznan.pl (J.C.); 2 Department of Human Nutrition, University of Warmia and Mazury in Olsztyn, Sloneczna 45F, 10-718 Olsztyn, Poland; joanna.kowalkowska@uwm.edu.pl (J.K.); lidia.wadolowska@uwm.edu.pl (L.W.); 3 Department of Organisation and Consumption Economics, Warsaw University of Life Sciences, Nowoursynowska 159 C, 02-776 Warsaw, Poland; marzena_jezewska_zychowicz@sggw.pl; 4 Department of Trade and Services, Gdynia Maritime University, Morska 81-87, 81-225 Gdynia, Poland; zielarz@am.gdynia.pl

**Keywords:** adolescents, dietary patterns, dietary restrictions, girls, principal component analysis

## Abstract

Dietary restraint is a commonly reported practice observed among young females. The practice remains controversial and can be interpreted as a beneficial self-regulating behavior or the opposite, an eating disorder that may have a detrimental effect on health. The aim of this short report was to investigate if dietary restrictions are associated with dietary patterns in a representative sample of Polish girls. Analyses were carried out on data from the Girls’ Eating Behavior and Health (GEBaHealth) study. The sample included 1107 girls, ranging in age from 13 to 21 years old. Restrictions regarding food quantities and selected food groups were assessed using a standardized interview. Dietary patterns were identified with Principal Component Analysis (PCA), based on dietary data collected with Food Frequency Questionnaires (FFQs). Logistic regression analysis was used to study the associations between self-reported restrictions and each dietary pattern. In the total sample, 30.5% of girls reported following some food restrictions. The most common restrictions regarded consumption of sugar and/or sweets (23.7%), high-fat foods (22.4%), and fats (21.3%). Girls who declared following any restrictions, restrictions in food quantity and restrictions in the consumption of sugar and/or sweets, high-fat foods, fats, cereals and/or bread and/or potatoes were more likely to adhere to the “fruit and vegetables” (considered pro-healthy) dietary pattern (adjusted odds ratios (ORs): 1.55, 95% CI: 1.14–2.12; 1.61, 95% CI: 1.17–2.21; 1.81, 95% CI: 1.30–2.52; 1.46, 95% CI: 1.04–2.06; 1.96, 95% CI: 1.38–2.80 and 3.25, 95% CI: 1.97–5.37, respectively), and less likely to adhere to the “fast foods and sweets” (unhealthy) and “traditional Polish” (rather unhealthy) patterns, compared to girls who declared no restrictions. Declared restrictions in the consumption of foods high in sugar, fat, and starch were observed in girls in the “fruit and vegetables” pattern and were uncommon in girls with unhealthy dietary patterns. Although cautious interpretation is needed when considering restrictions in the overall quantity of food consumed, the results indicate that dietary restrictions of sugar, high-fat foods, fats, and starch may be considered predictors of both pro-healthy and unhealthy dietary patterns in the population of Polish girls.

## 1. Introduction

Adolescence is a time of rapid growth; therefore, especially during this period, it is important to maintain adequate intake of food and nutrients for normal development and establish healthy dietary habits that will persist in later life [1]. However, the results of studies conducted in some countries indicate that a significant proportion of young people, particularly girls, use various quantitative and qualitative dietary restrictions [2]. This practice is controversial. Dietary restrictions may lead to unhealthy dietary habits and eating disorders, which can have a detrimental effect on nutritional status and health [3]. On the other hand, the diets of people in many developed countries are characterized by a high fat and sugar intake and low consumption of whole grains, fruit, and vegetables. Such diets can lead to the development of diet-related chronic diseases, including obesity, type 2 diabetes, cardiovascular diseases, and some cancers [4]. Therefore, moderate restraint in the consumption of foods undesirable in a healthy diet (e.g., energy-dense snacks) could be considered a beneficial and health promoting behavior.

One of the most commonly reported reasons for food restrictions is dissatisfaction with body weight and shape, which is more often observed in girls and women than in boys and men [5,6,7]. Adolescent girls often engage in weight management and dietary restriction hoping to achieve a socially desirable body shape [8].

Considering diet as a cluster of different dietary behaviors has been recently recommended; therefore, an analysis of dietary patterns, as opposed to individual foods or nutrients, has lately gained a great deal of interest in nutritional epidemiology [9]. Dietary patterns facilitate the study of the whole diet, recognizing that people consume foods in combination. This approach complements traditional methods of examining diet–health relationships that look at individual foods or nutrients [10].

To date, limited evidence has been reported describing the association between dietary restrictions and dietary patterns in adolescent girls. Therefore, the aim of this study was to investigate the associations between dietary restrictions and dietary patterns in a representative sample of Polish girls and young women.

## 2. Materials and Methods

### 2.1. Participants

Analyses were carried out on data from the Girls’ Eating Behaviour and Health (GEBaHealth) study; details regarding recruitment protocol and methodology have been previously reported [11]. In brief, 2104 girls aged 13–21 years old were contacted using the Universal Electronic System of Population Register (PESEL) database. Three-phase random sampling was applied to ensure the sample was representative for the Polish population. The computer-assisted personal interviewing (CAPI) technique was used to collect all data. Due to refusals, absence, disability, death, or other reasons, 47.4% of initially contacted girls (*n* = 997) did not take part in the study. As a result, the final sample included 1107 girls. Among these girls, missing or unreliable data were found to have misreported height (*n* = 3), weight (*n* = 15), and level of physical activity (*n* = 27); these data (but not participants) were removed from the analysis [11].

### 2.2. Ethical Approval

The Bioethics Committee of the Faculty of Medical Sciences, University of Warmia and Mazury in Olsztyn, approved the protocol of GEBaHealth study in 17 June 2010, Resolution No. 20/2010. Informed consent was provided by adult participants (18+ years) and/or under-aged participants’ parents or legal guardians (<18 years).

### 2.3. Dietary Restrictions

A standardized one-to-one interview (answers yes/no) at the respondent’s home was used to obtain data regarding dietary restrictions. Questions addressed 10 categories of restrictions: (1) restrictions in food quantity; (2) sugar and/or sweets; (3) high-fat foods; (4) fats; (5) cereals and/or bread and/or potatoes; (6) meats; (7) fish; (8) dairy products; (9) raw vegetables; and (10) raw fruit. Selection of the restrictions taken into consideration and the question structure were based on the results of our previous pilot study on 232 girls aged 13–21 years old (data not published).

### 2.4. Dietary Patterns

Dietary data collection and detailed methodology regarding dietary patterns identification have been previously described [11]. Briefly, data were collected using three food-frequency questionnaires: the Block Screening Questionnaire for Fruit/Vegetable/Fibre Intake (BSQFVF), the Block Screening Questionnaire for Fat Intake (BSQF), and the Food Intake Variety Questionnaire (FIVeQ). From all three questionnaires, data regarding 30 food items were collected and input into Principal Component Analysis (PCA)—with varimax rotation. Four dietary patterns were derived: “traditional Polish”, considered rather unhealthy (comprising of white bread, potatoes, red meats, margarine/butter, and fried chicken); “fruit and vegetables”, considered pro-healthy (comprising of salads, fruit, vegetables, and beans); “fast food and sweets”, considered unhealthy (comprising of fries, chips, burgers, ice-cream, pastries/cakes, salad dressings, sweets, and snacks); and “dairy and fats”, considered rather unhealthy (comprising of cheese, whole milk, margarine/butter, cereals and potatoes, and dairy products) (Table S1) [11]. 

### 2.5. Confounding Variables

Variables considered to be confounding were age, socioeconomic status, Body Mass Index (BMI), and physical activity level. Details regarding data collection and cut-off points for categorical variables have been previously described [11]. In brief, socioeconomic status was assessed by socioeconomic status index (SESI) based on four variables: mother’s education, father’s education, economic status, and description of household. BMI was calculated using self-reported weight and height and categorized in accordance with International Obesity Task Force (IOTF) standards [12]. Physical activity was assessed with International Physical Activity Questionnaire—Long (IPAQ-L) and expressed in Metabolic Equivalent of Task (MET)-minutes/week [13]. The level of physical activity was categorized as follows: low < 600, moderate = 600–2999, high ≥ 3000 MET-minutes/week, in accordance with IPAQ protocol [13].

### 2.6. Statistical Analysis

Girls in each of the dietary patterns were divided into three groups based on tertile distribution: 1st tertile (T1), 2nd tertile (T2), and 3rd tertile (T3); girls in T3 had the strongest adherence to the pattern. The percentage distribution of girls who reported dietary restrictions by dietary patterns was calculated. Associations between dietary restrictions (independent variables) and dietary patterns (dependent variables) were verified using logistic regression analysis. Odds ratios (ORs) represented the chances of the adherence to tertiles of each dietary pattern. The reference groups (OR = 1.00) were girls who declared not following the restrictions. Two models were created: crude and adjusted for age and SESI (as continuous variables) and for BMI and physical activity level (as categorical variables). Wald’s test was used to assess the significance of ORs. Tests of linear trends across increasing tertiles of dietary pattern adherence (for ORs) were calculated for each type of dietary restriction. *p*-value < 0.05 was considered significant for all tests. All analyses were carried out applying sample weights to adjust for non-response and missing data. Analyses were performed using STATISTICA software (version 10.0 PL; StatSoft Inc., Tulsa, OK, USA; StatSoft Polska, Krakow, Poland). 

## 3. Results

The main characteristics of study participants are summarized in Table 1. Nearly one-third (30.5%) of the study population declared following some restrictions in food consumption, and 27.9% declared following restrictions regarding the quantity of foods consumed. In the total sample, the most common restrictions regarded the consumption of sugar and/or sweets (23.7%), high-fat foods (22.4%), and fats (21.3%).

The associations between each of the dietary patterns and dietary restrictions are presented in Table 2. Crude ORs and adjusted ORs for age, socioeconomic status, BMI, and physical activity are shown. The chances of adherence to each dietary pattern reported in the text refer to significant ORs in the adjusted model.

Girls who declared following any restrictions or restrictions in quantity of food were over one and a half times more likely to adhere to the “fruit and vegetables” pattern (ORs: 1.55 and 1.61, respectively) and around 50% less likely to adhere to the “traditional Polish” (0.47 and 0.49), “fast food and sweets” (0.50 and 0.49), and “dairy and fats” (0.54 and 0.51) patterns. 

In terms of restrictions regarding selected food groups, girls who restricted either sugar/sweets, high-fat foods, fats, or cereals/bread/potatoes were more likely to adhere to the “fruit and vegetables” pattern (1.81, 1.46, 1.96, and 3.25, respectively) and less likely to adhere to the “traditional Polish” (0.44, 0.44, 0.37, and 0.22, respectively), “fast food and sweets” (0.38, 0.47, 0.48, and 0.56, respectively), and “dairy and fats” (0.41, 0.43, 0.41, and 0.38, respectively) patterns. Girls who restricted either meat, dairy products, or raw fruit were less likely to adhere to the “traditional Polish” (0.35, 0.50, and 0.23, respectively) and “dairy and fats” (0.60, 0.19, and 0.10, respectively) patterns. Girls who restricted raw vegetables consumptions were less likely to adhere to the “traditional Polish” and “fruit and vegetables” patterns (both ORs: 0.28). Associations with only one of the dietary patterns were found regarding restrictions in fish consumption. Girls who restricted fish were nearly 60% (0.42) less likely to adhere to the “traditional Polish” pattern.

## 4. Discussion

The results of our study show that, for Polish girls, dietary restrictions are associated with dietary patterns and that food restraint is a common practice, with over 30% of the study participants reporting this behavior. Quantitative restrictions as well as those related to foods undesirable in a healthy diet were associated with a pro-healthy dietary pattern. In parallel, girls who were not restricting their food intakes were more likely to have unhealthy dietary patterns.

In general, pro-healthy dietary behaviors were observed among girls in the “fruit and vegetables” dietary pattern—girls who had increased chances of frequent fruit, vegetables, and bean intakes [11]. Girls who adhered to this pattern reported restricting the overall quantity of food as well as foods high in fat, sugar, and starch. Avoidance of foods high in fat and sugar by adolescents who at the same time declare high intakes of fruit and vegetables has been previously reported [14]. Because all of these behaviors are in accordance with dietary recommendations, it can be speculated that girls from the “fruit and vegetables” pattern followed a range of complementing healthy behaviors, regardless of the motives behind them.

An interesting finding was that the odds of adherence to the “fruit and vegetables” pattern in girls who restricted starchy foods such as cereals, bread, and potatoes were greater (more than three times more likely) than in girls who restricted sugar and/or sweets, fats and high-fat foods (nearly two times more likely) when compared to girls with no restrictions. This finding could be explained by the increasing popularity of low-carbohydrate diets (e.g., Atkins, Zone, or Palaeolithic diet) strongly promoted in the media as a promising weight loss strategy [15]. Despite the controversies around the long-term efficacy and safety of low-carbohydrate diets [16], self-imposed restrictions of carbohydrates without medical supervision should be considered risky behavior among young people [17] and worth further research.

Unfavorable dietary behaviors were found in girls from patterns considered unhealthy—the “fast food and sweets” pattern—and rather unhealthy—“traditional Polish” and “dairy and fats.” As expected, girls with the highest adherence to the “fast food and sweets” pattern were less likely to restrict sugar and sweets (by 62%) and high-fat foods (by 53%), which could indicate little or no interest in the quality of their diet and perhaps strong preferences for highly palatable foods. Similar results were obtained by French et al. [18], who found that a higher frequency of fast food consumption was more prevalent in women with low dietary restraint. In fact, many studies have linked low-restraint with overweight and obesity; e.g., Kruger et al. [19] found that young females from the “low-restraint and high disinhibition” group had significantly higher BMI and body fat percentage than women from the “high-restraint and low-disinhibition” group. In that context, an absence of restrictions in terms of unhealthy foods should be considered an undesirable behavior. However, it must be highlighted that dietary restraint can be a dangerous practice and requires careful interpretation. In general, it is important what foods are being restricted and who is using those restrictions. For example, restricting the overall energy intake (rather than foods of a low nutritional value) or foods that are essential in a healthy diet may lead to unhealthy behaviors, such as the substitution of meals with snacks, alcohol, or smoking and can lead to episodes of binge eating, particularly in people with low self-esteem, social anxiety, and appearance concerns [20,21,22,23]. In this case, the maintenance of a balanced and varied diet would be a more appropriate dietary recommendation than introducing dietary restrictions. Lastly, our findings illustrate that the restrictions reported by the participants were coherent with the actual behavior (reflected in the identified dietary pattern), which may suggest that girls from our study provided honest answers regarding their dietary behaviors.

The associations between the “traditional Polish” pattern and dietary restrictions were not as straightforward. Girls from this pattern did not restrict the consumption of unhealthy foods but were also unlikely to report restricting the consumption of fish, dairy, and raw fruit and vegetables—foods that are desired in a balanced diet. However, a lack of restrictions does not equal an increased consumption of these foods and could simply be a sign of a lack of self-regulating behaviors presented by these girls. Observations of a similar nature were found within the “dairy and fats” pattern.

The main weaknesses of this study are the analysis of self-reported data and the certain limitations of the assessment tools used. Perhaps the study would benefit from the inclusion of total energy intake into the confounding variables. These data were not available to us due to the design of the questionnaires used in the study. However, because subjective energy intake is often misreported, adjustment for external predictors such as BMI and physical activity could be a better method than energy intake estimated from FFQs or another subjective dietary assessment method [24,25]. Lastly, we used a self-constructed questionnaire to assess dietary restrictions. It could be argued that a validated tool such as the Dutch Eating Behavior Questionnaire (DEBQ) [26] is a better tool to assess dietary restraint. However, the primary aim of this study was to find associations between following restrictions in the consumption of certain foods and dietary patterns, rather than focusing strictly on psychological aspects related to eating restraints. For this reason, we concluded that the use of a self-constructed questionnaire, previously tested in our pilot study is a more suitable tool.

The main strength is a relatively large, nationally representative sample of more than 1000 girls. Although our findings pertain only to young Polish females and should not be generalized to the wide population of adolescents, our study provides an interesting insight into dietary restrictions and their association with dietary patterns of adolescent girls.

## 5. Conclusions

Food restraint is a common practice among Polish girls, declared by over 30% of the study participants. Declared restrictions in the consumption of foods high in sugar, fat, and starch were observed in girls in the “fruit and vegetables” pattern and could be interpreted as an avoidance of foods that—if consumed in excess—are not desirable in a healthy diet; hence, the practice could be interpreted as a self-regulating and beneficial behavior. These restrictions were uncommon in girls with unhealthy dietary patterns, indicating that the self-regulating behavior among these girls may not be present. Although a cautious interpretation is needed when considering restrictions regarding the quantity of food consumed, the results indicate that dietary restrictions of sugar, high-fat foods, fats, and starch could be considered predictors of both pro-healthy and unhealthy dietary patterns in the population of Polish girls and young women.

## Figures and Tables

**Table 1:** Sample characteristics (%).

Variables	Total
Number of subjects †	1107
Age ^&^	17.3 ± 2.6
Age (years)	
13–15	29.5
16–18	33.1
19–21	37.4
Socioeconomic status ^a^	
Low	36.2
Medium	30.6
High	33.2
BMI category ^b^	
Thinnest grade 3	0
Thinnest grade 2	0.5
Thinnest grade 1	9.7
Normal weight	77.7
Overweight	10.5
Obesity	1.6
Physical activity ^c^	
Low	47.1
Moderate	50.9
High	2.0
Any restrictions in food consumption	30.5
Restriction on quantity of food consumption	27.9
Restrictions in consumption of:	
Sugar and/or sweets	23.7
High-fat foods	22.4
Fats	21.3
Cereals, bread and/or potatoes	12.0
Meats	11.3
Fish	6.2
Dairy products	5.5
Raw vegetables	2.5
Raw fruit	1.4

† Sample size may vary in each analysis due to missing data. All data adjusted for sample weights; ^&^ mean ± standard deviation. ^a^ Socioeconomic status categories based on tertile distribution. ^b^ BMI: body mass index (*n* = 1092). Weight status categories assigned in accordance with the International Obesity Task Force (IOTF) standards [12]; for girls 13–18 years old BMI age-sex-specific cut-offs were corresponding to the values at age 18; for girls > 18 years old in accordance with cut-offs for girls at age 18 (adults). ^c^ Physical activity classification: low: <600 Metabolic Equivalent of Task (MET)-minutes/week, moderate: 600–2999 MET-minutes/week, high: ≥3000 MET-minutes/week, in accordance with the International Physical Activity Questionnaire (IPAQ) protocol [13].

**Table 2:** Odds ratios (ORs (95% CI) of dietary patterns by dietary restrictions in Polish girls.

Variables		“Traditional Polish”	*p* for Trend		“Fruit & Vegetables”	*p* for Trend		“Fast Food & Sweets”	*p* for Trend		“Dairy & Fats”	*p* for Trend
	T1	T3		T1	T3		T1	T3		T1	T3	
Any restrictions in food consumption (ref.: without restrictions)												
OR crude (95% CI)	1.00	0.44 **** (0.33; 0.59)	ns	1.00	1.76 *** (1.31; 2.36)	ns	1.00	0.47 **** (0.35; 0.64)	↓	1.00	0.58 *** (0.43; 0.77)	ns
OR adjusted (95% CI)	1.00	0.47 **** (0.34; 0.64)	ns	1.00	1.55 ** (1.14; 2.12)	ns	1.00	0.50 **** (0.37; 0.68)	↓	1.00	0.54 **** (0.40; 0.73)	ns
Restriction on quantity of food consumption (ref.: without restrictions)												
OR crude (95% CI)	1.00	0.47 **** (0.35; 0.63)	ns	1.00	1.80 *** (1.34; 2.43)	ns	1.00	0.46 **** (0.34; 0.62)	↓	1.00	0.55 **** (0.40; 0.74)	ns
OR adjusted (95% CI)	1.00	0.49 **** (0.36; 0.68)	ns	1.00	1.61 ** (1.17; 2.21)	ns	1.00	0.49 **** (0.36; 0.67)	ns	1.00	0.51 **** (0.37; 0.70)	ns
Restrictions in consumption of (ref.: without restrictions):												
Sugar and/or sweets												
OR crude (95% CI)	1.00	0.41 **** (0.30; 0.56)	ns	1.00	2.05 **** (1.50; 2.82)	ns	1.00	0.37 **** (0.26; 0.51)	ns	1.00	0.43 **** (0.31; 0.60)	ns
OR adjusted (95% CI)	1.00	0.44 **** (0.32; 0.62)	ns	1.00	1.81 *** (1.30; 2.52)	ns	1.00	0.38 **** (0.27; 0.54)	ns	1.00	0.41 **** (0.29; 0.57)	ns
High-fat foods												
OR crude (95% CI)	1.00	0.40 **** (0.28; 0.55)	ns	1.00	1.73 *** (1.25; 2.40)	ns	1.00	0.44 **** (0.31; 0.61)	ns	1.00	0.46 **** (0.33; 0.63)	ns
OR adjusted (95% CI)	1.00	0.44 **** (0.31; 0.62)	ns	1.00	1.46 * (1.04; 2.06)	ns	1.00	0.47 **** (0.33; 0.66)	ns	1.00	0.43 **** (0.31; 0.61)	ns
Fats												
OR crude (95% CI)	1.00	0.32 **** (0.23; 0.45)	ns	1.00	2.30 **** (1.65; 3.22)	ns	1.00	0.45 **** (0.32; 0.63)	ns	1.00	0.42 **** (0.30; 0.59)	ns
OR adjusted (95% CI)	1.00	0.37 **** (0.26; 0.54)	ns	1.00	1.96 *** (1.38; 2.80)	ns	1.00	0.48 **** (0.34; 0.69)	ns	1.00	0.41 **** (0.28; 0.58)	ns
Cereals and/or bread and/or potatoes												
OR crude (95% CI)	1.00	0.20 **** (0.13; 0.33)	ns	1.00	3.56 **** (2.21; 5.72)	ns	1.00	0.46 *** (0.29; 0.70)	ns	1.00	0.35 **** (0.23; 0.54)	ns
OR adjusted (95% CI)	1.00	0.22 **** (0.13; 0.37)	ns	1.00	3.25 **** (1.97; 5.37)	ns	1.00	0.56 * (0.35; 0.87)	ns	1.00	0.38 **** (0.24; 0.59)	ns
Meats												
OR crude (95% CI)	1.00	0.34 **** (0.22; 0.52)	ns	1.00	1.47 (0.97; 2.23)	ns	1.00	0.62 * (0.40; 0.94)	ns	1.00	0.69 (0.45; 1.07)	↓
OR adjusted (95% CI)	1.00	0.35 **** (0.22; 0.56)	ns	1.00	1.38 (0.89; 2.14)	ns	1.00	0.69 (0.45; 1.07)	ns	1.00	0.60 * (0.38; 0.95)	ns
Fish												
OR crude (95% CI)	1.00	0.45 ** (0.26; 0.79)	ns	1.00	0.86 (0.50; 1.50)	ns	1.00	0.85 (0.50; 1.45)	ns	1.00	0.86 (0.50; 1.48)	ns
OR adjusted (95% CI)	1.00	0.42 ** (0.24; 0.75)	ns	1.00	0.90 (0.51; 1.59)	ns	1.00	0.94 (0.54; 1.63)	ns	1.00	0.69 (0.39; 1.23)	ns
Dairy products												
OR crude (95% CI)	1.00	0.55 (0.30; 1.01)	ns	1.00	1.26 (0.70; 2.27)	ns	1.00	0.66 (0.38; 1.16)	ns	1.00	0.21 **** (0.11; 0.43)	ns
OR adjusted (95% CI)	1.00	0.50 * (0.27; 0.94)	ns	1.00	1.11 (0.60; 2.06)	ns	1.00	0.72 (0.40; 1.28)	ns	1.00	0.19 **** (0.09; 0.38)	ns
Raw vegetables												
OR crude (95% CI)	1.00	0.31 * (0.12; 0.79)	ns	1.00	0.29 ** (0.12; 0.74)	ns	1.00	0.63 (0.28; 1.41)	ns	1.00	0.59 (0.24; 1.43)	ns
OR adjusted (95% CI)	1.00	0.28 ** (0.11; 0.73)	ns	1.00	0.28 ** (0.11; 0.73)	ns	1.00	0.63 (0.28; 1.44)	ns	1.00	0.51 (0.21; 1.28)	ns
Raw fruit												
OR crude (95% CI)	1.00	0.23 * (0.07; 0.84)	ns	1.00	0.62 (0.20; 1.94)	ns	1.00	0.28 (0.08; 1.03)	ns	1.00	0.21 * (0.05; 0.99)	ns
OR adjusted (95% CI)	1.00	0.23 * (0.06; 0.87)	ns	1.00	0.68 (0.19; 2.43)	ns	1.00	0.36 (0.09; 1.37)	ns	1.00	0.10 * (0.01; 0.84)	ns

All data adjusted for sample weights. ORs were adjusted for: age (continuous variable in years), socioeconomic status (continuous variable in points measured as socioeconomic status index (SESI), which was calculated from four single variables: mother’s education, father’s education, economic status, description of household), BMI (categorical variable with six categories: for girls 13–18 years old according to age-sex-specific BMI cut-offs; for girls > 18 years old according to cut-offs for girls at age 18 [12]) and physical activity (categorical variable with three categories: <600, 600–2999, ≥3000 MET-minutes/week [13]). Statistically significant: * *p* < 0.05, ** *p* < 0.01, *** *p* < 0.001, **** *p* < 0.0001 (Wald’s test). ↓ decreasing linear trend at *p* < 0.05. ns—statistically insignificant.

## Supplementary Materials

The following are available online at http://www.mdpi.com/2072-6643/8/12/796/s1, Table S1: Components of dietary patterns.
