# Supplementary material for: Self-Reported Dietary Restrictions and Dietary Patterns in Polish Girls: A Short Research Report (GEBaHealth Study)
Source: Nutrients. 2016 Dec 19;8(12):796. doi: 10.3390/nu8120796 (PMC5188451; doi:10.3390/nu8120796)
Supplement: Supplementary file 1 [file nutrients-08-00796-s001.docx]

Supplementary Materials: Self-Reported Dietary Restrictions and Dietary Patterns in Polish Girls: A Short Research Report (GEBaHealth Study)

Grzegorz Galinski, Marta Lonnie, Joanna Kowalkowska, Lidia Wadolowska, Jolanta Czarnocinska, Marzena Jezewska-Zychowicz and Ewa Babicz-Zielinska

**Table S1.** Components of dietary patterns.

| **Dietary Pattern** | **Dietary Pattern Characteristics (Factor Loading)** | | | |
| --- | --- | --- | --- | --- |
|  | **Food Frequency Consumption ^a^** | | **Food Intake Variety By Food Groups ^b^** | |
| “Traditional Polish” | White bread (including biscuits, muffins)  Potatoes  Red meats  Margarine or butter  Fried chicken  Wholegrain bread | (0.65)  (0.52)  (0.51)  (0.45)  (0.42)  (−0.48) | Meats, fish and eggs  Fats | (0.60)  (0.45) |
| “Fruit & vegetables” | Green salad  Fruit (without juices)  Prepared vegetables  Beans | (0.57)  (0.55)  (0.55)  (0.45) | Vegetables  Fruit | (0.60)  (0.54) |
| “Fast food & sweets” | French fries or potato chips or corn chips or popcorn  Hamburgers or cheeseburgers  Ice cream  Doughnuts or pastries or cakes or cookies  Salad dressings or mayonnaise (not diet) | (0.71)  (0.60)  (0.52)  (0.50)  (0.42) | Sweets and snacks | (0.47) |
| “Dairy & fats” | Margarine or butter  Cheese or cheese spread  Whole milk | (0.45)  (0.54)  (0.49) | Fats  Cereals and potatoes  Dairy products | (0.43)  (0.56)  (0.54) |

^a^ Food frequency consumption was expressed in points (range 0–4 points). ^b^ Food intake variety was expressed in foods consumed per week (with ranges from 0–4 to 0–14 foods/week).
